# *Jasminanthes xuanlienensis* (Apocynaceae, Asclepiadoideae), a new species from Vietnam

**DOI:** 10.3897/phytokeys.69.9272

**Published:** 2016-08-18

**Authors:** The Bach Tran, Do Van Hai, Bui Thu Ha, Michele Rodda

**Affiliations:** 1 Department of Botany, Institute of Ecology and Biological Resources, Vietnam Academy of Science and Technology, 18 Hoang Quoc Viet, Cau Giay, Hanoi, Vietnam; 2 Hanoi National University of Education, 136, Xuan Thuy Street-Cau Giay District, Hanoi, Vietnam; 3 The Herbarium, Singapore Botanic Gardens, 1 Cluny Road, 259569 Singapore

**Keywords:** Marsdenieae, China, Xuan Lien National Park

## Abstract

*Jasminanthes xuanlienensis* (Apocynaceae, Asclepiadoideae), a new species from Vietnam is described, illustrated and compared with its five congeners. *Jasminanthes xuanlienensis* differs distinctly from congeners by the longer peduncles (14–18 cm vs. 4 cm at most in *Jasminanthes pilosa* and *Jasminanthes saxatilis*, salmon-pink color of the inner corolla lobes (white or greenish in the other species), and corolla tube length (12.0–14.5 mm vs. shorter or longer in congeners).

## Introduction

*Jasminanthes* Blume (Apocynaceae, Asclepiadoideae, Marsdenieae) is a small Old World genus of six species (Endress et al. 2014). Its type species, *Jasminanthes suaveolens* Blume, is from Java. However, the centre of diversity of *Jasminanthes* is mainland Asia, particularly China, where the remaining congeners are found (Gilbert et al. 1995, Li et al. 1995). *Jasminanthes* was considered to be a large-flowered *Marsdenia* R.Br. by Forster (1995). However, the very broad circumscription of *Marsdenia* adopted by Forster is not based on phylogenetic evidence and has not been generally accepted in later works. For instance *Gymnema*, also considered congeneric with *Marsdenia* by Forster (1995), was retained in floristic works such as Jagtap and Singh (1999) and Watson (1999). The phylogeny of Surveswaran et al. (2014) does not address whether *Gymnema* should be merged with *Marsdenia*. The purpose of the present paper is limited to validate a new taxon and we believe that its correct placement is in *Jasminanthes*. *Jasminanthes* is separated from *Marsdenia* based on its large flowers (>15 mm long) with salverform corolla and by its inconspicuous to absent staminal corona. In contrast, *Marsdenia* usually has much smaller flowers (<10 mm long) with urceolate or rotate corolla and conspicuous staminal corona. If molecular evidence later proves that *Jasminanthes* is indeed to be considered within a broadly circumscribed *Marsdenia*, a new combination will be required.

In Vietnam, no species of *Jasminanthes* have been recorded to date (Ho 1993, Tran 2005). However, recent fieldwork in Xuan Lien National Park, Vietnam, yielded a collection of *Jasminanthes* that was identified as a new species based upon comparison with the known species in the literature and specimens at BK, BKF, BM, HN, HNU, HNPM, IBK, IBSC, K, KUN, KYO, P, SING, TI, TO, TUT, and VNM. Here we describe the new species and provide a detailed table of character differences among the species along with a key to the species of *Jasminanthes*.

## Species treatment

### Jasminanthes xuanlienensis

Taxon classificationPlantaeGentianalesApocynaceae

T.B.Tran & Rodda sp. nov.

urn:lsid:ipni.org:names:60472843-2

Fig. 1

#### Type.

Vietnam. Thanh Hoa province: Xuan Lien National Park, 720 m, N19°59'14.6", E104°59'49.1", 22 April 2013, *Do Van Hai et al. XL 904* (HN, holotype; HN, isotype).

#### Description.

*Liana* large, up to 10 m long. *Stems* glabrous, 1.7–2 mm in diameter; *internodes* 21–23 cm long. *Leaves* opposite; *petiole* 1.6–2.6 cm long, 1.5–2 mm in diameter, pubescent with spreading trichomes, *lamina* variable in shape, elliptic (ovate), 11–15 × 4.1–6.8 cm, with many black spots when dry; adaxial surface glabrous except sparsely pubescent base with spreading trichomes, basal colleters 1–3 each lamina base, ovoid; abaxial surface pubescent, with spreading trichomes; base round to acuminate; apex acuminate with a caudate tip 0.8–1.5 cm long; lateral veins 9–13 pairs. *Inflorescences* extra-axillary, simple or dichotomous, umbelliform, up to 30-flowered; *peduncle* 14–18 cm long, 1.3–1.7 mm in diameter, pubescent, trichomes spreading to retrorse. *Pedicel* 8.5–11.0 mm long, 0.2–0.5 mm in diameter, pubescent with spreading trichomes. *Flower bud* just before anthesis fusiform, 10.7–13.0 mm long, 1.9–2.9 mm in diameter, apex acuminate, base bulbous. *Calyx* sepals 5, free; *lobes* triangular-linear, apex acuminate, 2.5–2.6 × 0.6–1.0 mm, adaxial surface glabrous, abaxial surface pubescent with spreading trichomes; *colleters* occurring singly between the sepals, ovoid-conical, 0.2–0.3 × 0.1–0.2 mm. *Corolla* salverform, tube 12.0–14.5 mm long, adaxially with 5 pairs of longitudinal lines of retrorse trichomes, abaxially glabrous, throat pubescent, eglandular trichomes 0.36–0.70(–1.34) mm long, lobes elliptic-ovate to triangular, 7.0–8.0 × 3.0–3.6 mm, adaxial surface salmon-pink, abaxial surface greenish-white. *Corona* staminal; corona lobes arrow-shaped, much reduced and attached to gynostegium lengthwise, c. 1.8 mm high, c. 0.8 mm wide, glabrous. *Guide rails* 1.7–2.0 mm long. *Gynostegium* 3.5–4.0 mm tall, 1.8–2.0 mm wide. *Pollinia* erect, oblong, 0.49–0.58 mm × 0.2–0.23 mm; *caudicle* 0.12–0.13 mm × 0.05–0.06 mm; *corpusculum* rhomboid, 0.2–0.23 × 0.10–0.11 mm. *Anther appendages* erect, covering the style head, 1.0–1.2 × 0.7–0.9 mm. *Style head* conical with a round tip, 0.8–1.0 mm high. *Ovary* bi-carpellate, carpels ovoid, c. 1.60 mm long, 1.10 mm wide at the base. *Fruits* and *seeds* not observed.

**Figure 1.**
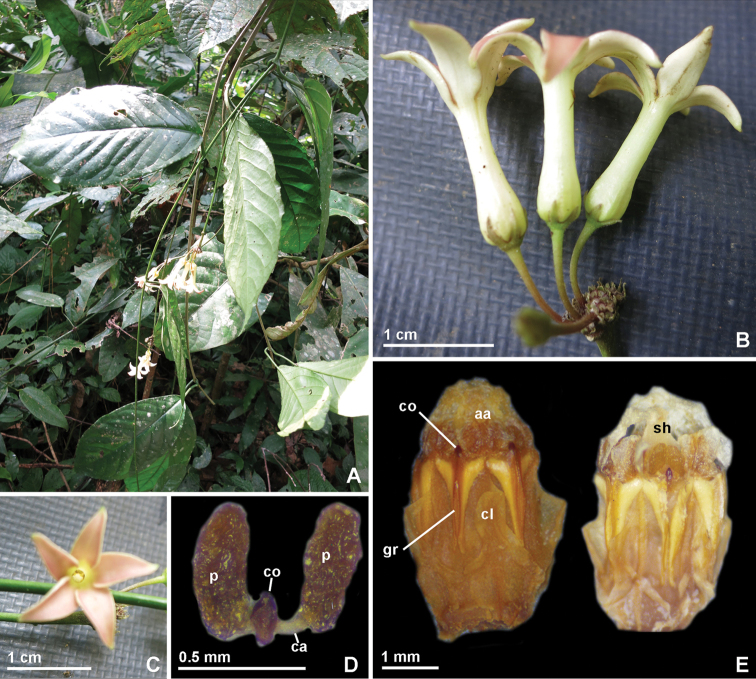
*Jasminanthes xuanlienensis* T.B.Tran & Rodda. **A** Flowering branch in the habitat of the type locality (22 April 2013) **B** Inflorescence **C** Flower, top view **D** Pollinarium **E** Gynostegium and staminal corona. aa: anther appendage; ca: caudicle; cl: corona lobe; co: corpusculum; gr: guide rail; p: pollinium; sh: style head. (Photographs by T.B. Tran, photo edit by M. Rodda)

#### Etymology.

The species is named after the type locality, Xuan Lien National Park, Thanh Hoa province, in northern Vietnam.

#### Distribution and ecology.

*Jasminanthes xuanlienensis* was found growing in primary evergreen forests on limestone soil of Xuan Lien National Park. It was collected in flower in April. Plants observed growing in the vicinity include *Piper acreanum* C.DC., *Beccarinda tonkinensis* (Pellegr.) B.L.Burtt, *Sarcosperma kachinense* (King & Prain) Exell, *Hoya vercillata* (Vahl) G.Don, and *Alangium salviifolium* (L.f.) Wangerin.

#### Conservation status.

*Jasminanthes xuanlienensis* is known only from the type locality, an area still poorly known botanically; its preliminary conservation status is therefore Data Deficient (DD; IUCN 2014).

#### Notes.

The six species of *Jasminanthes* are clearly distinguishable from one another based on morphological characters (Table 1). The new species can be easily separated from all *Jasminanthes* species as it has very long peduncles (14–18 cm long), while the longest peduncles observed in other species are those of *Jasminanthes pilosa* (Kerr) W.D.Stevens & P.T.Li and *Jasminanthes saxatilis* (Tsiang & P.T.Li) W.D.Stevens & P.T.Li), which reach 4 cm long. *Jasminanthes xuanlienensis* has unique pink corolla lobes whereas the other species have white corollas, or greenish corollas in *Jasminanthes saxatilis*.

**Table 1.** Morphological comparision of *Jasminanthes* species.

Characters	*Jasminanthes xuanlienensis*	*Jasminanthes chunii*	*Jasminanthes mucronata*	*Jasminanthes pilosa*	*Jasminanthes saxatilis*	*Jasminanthes suaveolens*
**Lamina shape**	elliptic (ovate)	ovate	ovate-oblong	oblong-ovate	elliptic-lanceolate	elliptic-lanceolate
**Lamina base**	acuminate	cordate-subcordate	cordate-subcordate	cordate	acute	acute to round
**Lamina apex**	caudate	acute-cuspidate	acuminate-short caudate	shortly acuminate	acuminate-caudate	acuminate-cuspidate
**Lateral veins (pairs)**	9–13	4–7	5–7	7–10	5–6	5–8
**Length of petiole (cm)**	1.6–2.6	1–1.5(–2)	1.5–3	1–4.5	1.3–1.8	0.7–1.2
**Number of flowers**	up to 30	up to 12	2–4(–9)	5–10	c. 10	c. 10
**Length of peduncle (cm)**	14–18	(0.5–)1–1.5 cm	1–2	2–4 cm	3–4	0.5–1
**Length of pedicel (cm)**	0.85–1.1	1–1.3	1–3	0.7–1	0.8–1	0.7–1
**Shape of sepal**	triangular	oblong	elliptic-oblong	oblong-lanceolate	triangular	triangular
**Size of sepal (mm)**	2.5–2.6 × 0.6–1	5–6 × c. 2	7–8 × 3–4	18–30 × 4–8 mm	c. 3 × 1	5.5–8 × 2–3.5
**Corolla lobe colour**	adaxially salmon-pink, abaxially greenish-white	white	white	white	greenish	white
**Length of corolla tube (mm)**	12.0–14.5	7–9	c. 15	40–50	6–8	9–11
**Size of corolla lobe (mm)**	7.0–8.0 × 3.0–3.6	6–8 × 4–5	15–17 × 6–8	25–30 × 6–8	c. 10 × 3	7.0–9.0 × 1.0–1.5

### Key to the species of *Jasminanthes*

**Table:** 

1	Peduncle >10 cm long; corolla salmon, pink adaxially	***Jasminanthes xuanlienensis***
–	Peduncle <5 cm long; corolla white or greenish adaxially	**2**
2	Sepals >15 mm long; corolla tube >35 mm long	***Jasminanthes pilosa***
–	Sepals <10 mm long; corolla tube >10 mm long	**3**
3	Corolla tube >13 mm long, lobes >14 mm long	***Jasminanthes mucronata***
–	Corolla tube <11 mm long, corolla lobes <10 mm long	**4**
4	Lamina ovate, base cordate or subcordate	***Jasminanthes chunii***
–	Lamina elliptic-lanceolate, base acute to round	**5**
5	Sepals <3.5 mm long; peduncle >3 cm long	***Jasminanthes saxatilis***
–	Sepals >5 mm long; peduncle <1.5 cm long	***Jasminanthes suaveolens***

## Supplementary Material

XML Treatment for Jasminanthes xuanlienensis
